# A case report of wrist synovial infection due to *Mycobacterium jacuzzii*, Iran

**DOI:** 10.1186/s12879-020-05385-w

**Published:** 2020-09-16

**Authors:** Fatemeh Sakhaee, Morteza Masoumi, Farzam Vaziri, Seyed Davar Siadat, Abolfazl Fateh

**Affiliations:** 1 Department of Mycobacteriology and Pulmonary Research, Pasteur Institute of Iran, Tehran, Iran; 2 Microbiology Research Center (MRC), Pasteur Institute of Iran, Tehran, Iran

**Keywords:** *Mycobacterium jacuzzii*, Synovial infection, Iran

## Abstract

**Background:**

*Mycobacterium jacuzzii (M. jacuzzii)* was first isolated in 2003 by insertion of breast implants in Tel Aviv, Israel. In this case report, we describe our experience in detection of *M. jacuzzii* using phenotypic and genotypic test of wrist synovial sample.

**Case presentation:**

A 73-year-old woman complained of pain and swelling in the right wrist for 4 months. Her body temperature was 37–38 °C, and symptoms, such as pain, swelling, and some movement limitation, were reported. Clinical laboratory parameters showed an elevated C-reactive protein (CRP) level, erythrocyte sedimentation rate (ESR), and white blood cells (WBC) count. The sequences of *hsp65, rpoB, 16S rDNA*, and *sodA* genes indicated very high homology to *M. jacuzzii*.

**Conclusion:**

We report a case of synovial infection caused by *M. jacuzzii* in a patient with severe wrist pain in Iran, who was treated with amikacin, levofloxacin, and ethambutol. The outcomes of treatment after 8 months were positive, and no recurrence of infection was reported in the patient.

## Background

In the past several years, the outbreak of human diseases associated with nontuberculous mycobacteria (NTM) has been increasing. The cause of this increase is probably multifactorial, depending on the host, nature of infectious agent, and their interactions [1]. Due to the redundancy of tissue and synovial fluid and a higher probability of penetrating injury, NTM tenosynovitis happens most frequently in the hand and wrist. Usually, slowly growing mycobacteria species, especially *Mycobacterium (M.) marinum*, are involved [2]. In the present study, we report for the first time the isolation of *M. jacuzzii* from wrist synovial in a 73-year-old woman patient.

## Case presentation

A 73-year-old woman complained of pain and swelling in the right wrist for 4 months. Her body temperature was 37–38 °C, and symptoms, such as pain, swelling, and some movement limitation, were reported. Moreover, clinical laboratory parameters showed an elevated C-reactive protein (CRP) level (24 mg/L), erythrocyte sedimentation rate (ESR) (51 mm/h), and white blood cells (WBC) count (121,000 cells per cubic millimeter). Also, the rheumatoid factor and antinuclear antibody were negative. No history of trauma, diabetes, or immunosuppressive drug consumption was reported. Meanwhile, the patient received a local injection of methylprednisolone (20 mg daily/2 weeks). His swollen partially resolved but worsened again.

The synovial fluid sample was aspirated from the wrist and sent to Pasteur Institute of Iran in January 2019 for evaluating the presence of *Mycobacterium (M.) tuberculosis*. The results of smear test were positive for acid-fast bacillus (AFB).

A needle biopsy was performed for pathological and microbiological tests. After tissue evaluation, the synovium, containing epithelioid cells, fibrotic changes, and numerous noncaseating granulomas, was obviously thickened due to chronic inflammation. Ziehl-Neelsen staining indicated AFB in the tissue.

A synovial fluid sample was cultured on the Lowenstein-Jensen medium. After 5 days, the results indicated rapidly-growing mycobacteria (RGM) with smooth*,* small, and non-pigmented colonies. The results of biochemical tests were negative for three-day arylsulfatase growth at 45 °C, niacin accumulation, and nitrate reductase, while they were positive for growth on MacConkey agar without crystal violet, heat-resistant catalase, and iron uptake tests.

**Fig. 1:**
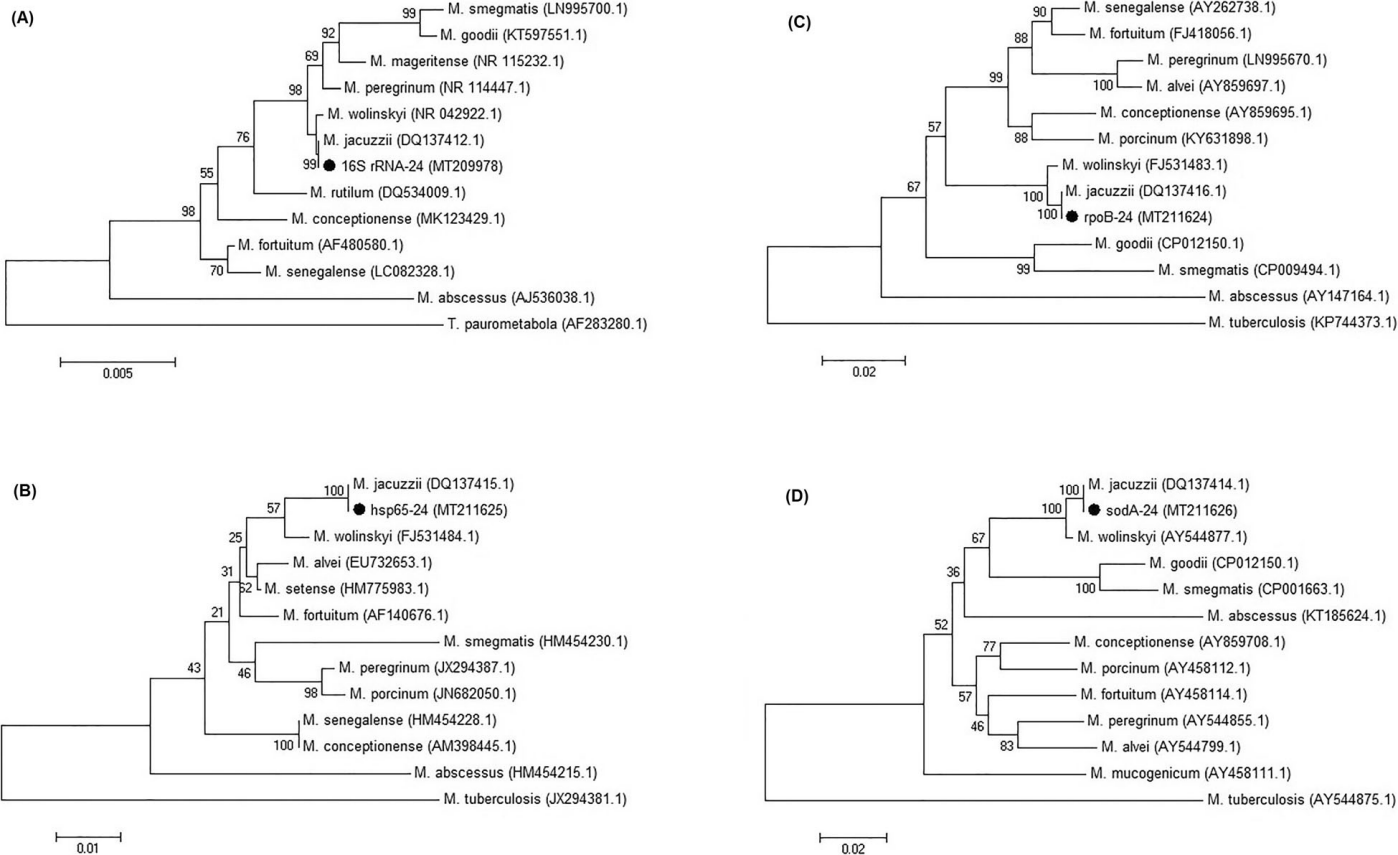
Neighbor-joining tree of the *16S rDNA* (**a**), *hsp65* (**b**), *rpoB* (**c**), and *sodA* (**d**) genes of mycobacteria and our isolate. Outgroup for *hsp65*/*rpoB*/ *sodA* genes and *16S rDNA* gene were *Mycobacterium tuberculosis* and *Tsukamurella paurometabola*, respectively. The GenBank accession numbers are given in parentheses for the reference sequences. Bootstrap values are represented on branch nodes. The nucleotide sequences identified in this study were submitted to GenBank under the accession numbers, MT209978, MT211625, MT211624, and MT211626 for *16S rDNA*, *hsp65*, *rpoB*, and *sodA* genes, respectively

The multilocus sequence analysis was also performed using partial *hsp65*, *rpoB*, *sodA,* and full *16S rDNA* genes, as previously described [2–4]. The results of biochemical tests indicated homology to *M. wolinskyi,* while the sequences of *hsp65* (441-bp)*, rpoB* (750-bp)*, 16S rDNA* (~ 1500-bp), and *sodA* (524-bp) genes indicated very high homology to *M. jacuzzii* (Fig. 1), as previously shown [5].

The drug susceptibility pattern (DSP) test was performed according to the Clinical and Laboratory Standards Institute (CLSI) guidelines [6]. The results indicated that the *M. jacuzzii* was extremely resistant to isoniazid, rifampin, clarithromycin, sulfamethoxazole-trimethoprim, capreomycin, cycloserine, tobramycin, cefoxitin, imipenem and streptomycin and highly susceptible to amikacin, levofloxacin, doxycycline, ofloxacin, ethambutol, and ciprofloxacin.

Antimicrobial therapy was performed according to the in vitro susceptibility test, and the patient was treated with amikacin, levofloxacin and ethambutol for 3 months. She recovered slowly, while some wrist swelling was still observed; however, pain and swelling significantly reduced. Eight months after anti-mycobacterial therapy, the patient showed complete recovery, and CRP, ESR, and WBC count were normal.

## Discussion and conclusion

So far, *M. jacuzzii* has been only identified in patients with a history of breast surgery, involving implants from August to November 2003 at a single medical center in Tel Aviv, Israel. In this study, the prevalence of surgical site infection caused by *M. jacuzzii* following breast implantation was determined. The infectious agent was identified during surgery by an asymptomatic surgeon, who had collected the isolate from his whirlpool and suggested that this bacterium might be transmitted through human-to-human contact [5].

To the best of our knowledge, synovial infection caused by *M. jacuzzii* has not been previously reported. In this regard, Olsen et al. evaluated 66 cases of synovitis caused by NTM, including *M. avium* complex*, M. terrae, M. nonchromogenicum, M. malmoense, M. haemophilum, and M. xenopi*. The majority of these patients had an immunodeficiency and a history of wound, trauma, or invasive medical procedure around the joints [7]. The studied patient had received methylprednisolone, but she did not have any history of trauma or surgery on her wrist; therefore, we were unable to identify the entry route of the pathogen. However, this may not be an unusual occurrence, as in only 39% of mycobacterial synovitis cases, the source of inoculum can be identified for the infectious agent [7].

Mycobacterial synovitis therapy generally includes surgical excision and antibiotic administration [7]. However, due to the lack of clinical experience and under-reporting of this isolate, the optimal treatment for *M. jacuzzii* infection has been standardized, including the duration of therapy, anti-mycobacterial drugs, and combination therapy. Only one study has explained the clinical course of *M. jacuzzii* infection. It was found that the removal of implants, total capsulectomy, and excision of all granulated tissue were effective treatments, but did not involve antimicrobial therapy [5].

Since only a limited number of anti-mycobacterial agents exhibit in vitro activity against *M. jacuzzii*, a combination therapy was initiated in our case with drugs to which the bacterium was susceptible, including amikacin, levofloxacin, and ethambutol. The outcomes of treatment after 8 months were positive, and no recurrence of infection was reported in the patient. In addition to other well-known NTM, our findings suggest that *M. jacuzzii* strain should be considered as a possible cause of synovitis.

## Data Availability

All the data supporting the findings is contained within the manuscript. Sequence data of this organism that support the findings of this study have also been deposited in GenBank database with the accession number MT209978 for *16S rDNA* gene (https://www.ncbi.nlm.nih.gov/nuccore/MT209978) and  MT211624 (https://www.ncbi.nlm.nih.gov/nuccore/MT211624), MT211625 (https://www.ncbi.nlm.nih.gov/nuccore/MT211625), and MT211626 (https://www.ncbi.nlm.nih.gov/nuccore/MT211626) for *rpoB*, *hsp65*, and *sodA* genes, respectively.

## Abbreviations

*M. jacuzzii*: *Mycobacterium jacuzzii*
CRP: C-reactive protein
ESR: Erythrocyte sedimentation
WBC: White blood cells
NTM: Nontuberculous mycobacteria
AFB: Acid-fast bacillus
RGM: Rapidly-growing mycobacteria
DST: Drug susceptibility pattern
CLSI: Clinical and Laboratory Standards Institute
